# Compost preparation, chemical analyses and users’ perception in the utilization of water hyacinth, Ethiopia

**DOI:** 10.1186/s13065-022-00851-9

**Published:** 2022-07-30

**Authors:** Dessie Tibebe, Kehali Jembere, Addisu Kidie, Marelign Adugna, Teferi Alem, Gizachew Teshome

**Affiliations:** 1 College of Natural and Computational Sciences, University of Gondar, P. O. box 196, Gondar, Ethiopia; 2 College of Agriculture and Environmental Sciences, University of Gondar, P. O. box 196, Gondar, Ethiopia

**Keywords:** Compost, Water hyacinth, Lake Tana, Treatment, Heavy metal

## Abstract

Lake Tana is the largest freshwater body in Ethiopia. Currently, the lake has been facing alarming environmental degradation and loss of biodiversity due to the invasion of water hyacinth. Although the weed is invasive, it can be converted into various benefits. Hence, this study was conducted in North Eastern Lake Tana, Sheha Gomengie Kebele. The main objective is compost preparation in terms of its drying periods, analyses, and user perception. Physicochemical and nutrient analyses were performed according to the standard procedures. Acid digestion was used for heavy metal analyses. From the result, the pH measurements ranged from 7.619 ± 0.195 to 7.719 ± 0.261, and the moisture content ranged from 38.712 ± 0.680 to 49.60 ± 9.06%. The mean electrical conductivity (EC) values of all treatments of matured compost ranged from 2.780 ± 0.542 to 3.51 ± 0.971 ds/m. The TN values of the matured compost ranged from 0.420 ± 0.379 to 0.754 ± 0.194 on a dry weight basis. The overall mean values of the C:N ratio for all the treatments were 11.60 which is within an acceptable range. A high amount of available P concentrations was observed in all compost treatments which ranged from 2.740 ± 0.190 to 2.940 ± 1.410 g/kg. Moreover, the concentrations of heavy metals in all treatments were below the permissible limit of different agencies and there was also no significant difference in the mean values of analysis of variance at (*P* < 0.05). Therefore, the prepared compost can be recommended for better agricultural purposes. Considering users’ understanding of compost preparation as an opportunity, converting WH into compost is promising in terms of its rich supply and the possibility of preparing in the dry season where labor is abundant. Therefore, it can be one way of sustainably reducing WH adverse effects on the Lakeshore.

## Introduction

The worldwide distribution of water hyacinth (Eichhornia crassipes) commonly considered an aquatic weed, has become a pushy and luxurious aquatic problem damaging the environment, irrigation system, and crops. Water hyacinth is a type of weed that grows very fast. The growth of water hyacinth can be 1.9% a day with 0.3–0.5 m in length [1–3].

Lake Tana is the largest freshwater body in Ethiopia, which constitutes 50% of the country’s fresh water reserve and has substantial socio-ecological importance. Despite its ecological and economic importance both locally and globally, Lake Tana has been facing alarming environmental degradation and loss of biodiversity due to human pressure, land-use changes and climate change related problems. The lake experiences ecological disorder and widespread eutrophication, which causes the death of fish, oxygen depletion, and the growth of aquatic weeds such as water hyacinth (WH) [4]. Water hyacinth was first recognized by the International Union for Conservation of Nature (IUCN) as one of the 100 maximum harsh destructive species [5, 6]. The Ethiopian government has attempted to control the weed by mobilizing affected farming communities to remove it using manual labor. Currently, a mechanical harvester machine is used to chop, harvest, and dispose of the weed. These fragmented efforts are found inefficient in controlling the spread of the weed and its economic, social, and ecological consequences [5, 6].

Methods to control WH have been proposed, such as biological and chemical eradication, and physical removal by manpower or machine. Each method has its own benefits and drawbacks [7]. Given the economic and environmental problems involved, the Ethiopian government has attempted to control the weed by mobilizing affected farming communities to remove it using manual labor. Currently, a mechanical harvester machine is used to chop, harvest, and dispose of the weed. The WH biomass is usually burned or thrown onto the banks in piles, which results in it rotting. When this happens, methane gas, which contributes to climate change, is released. These fragmented efforts are found inefficient in controlling the spread of the weed and its economic, social, and ecological consequences. So far, there is no attempt to transform the WH biomass into value-generating products such as organic fertilizer (compost) for agricultural inputs. So far in Ethiopia, there was no attempt to transform the WH biomass into value-generating products such as organic fertilizer (compost) and to use them as an input for agricultural activities. Therefore, utilization of this weed for a different purpose is one of the recommended solutions for reducing the excessive growth and related problems of water hyacinth. Thus, this research project was designed to contribute to long-term water hyacinth management through the utilization of water hyacinth biomass for compost preparations and its application. Therefore, this study aimed to produce quality compost, characterize the chemical composition of the prepared compost, and develop users’ perception to utilize WH in valuable products.

## Materials and methods

### Description of the study area

Lake Tana is found at 100 58′–120 47′ N latitude and 360 45′–380 14′ E longitude. The water hyacinth is highly infested at the coast of Dera, Fogera, Libo kemkem, Gondar Zuria, and Gorgora woredas. There were about 5396 hectares of water hyacinth coverage found in all woredas [8–10].

**Fig. 1:**
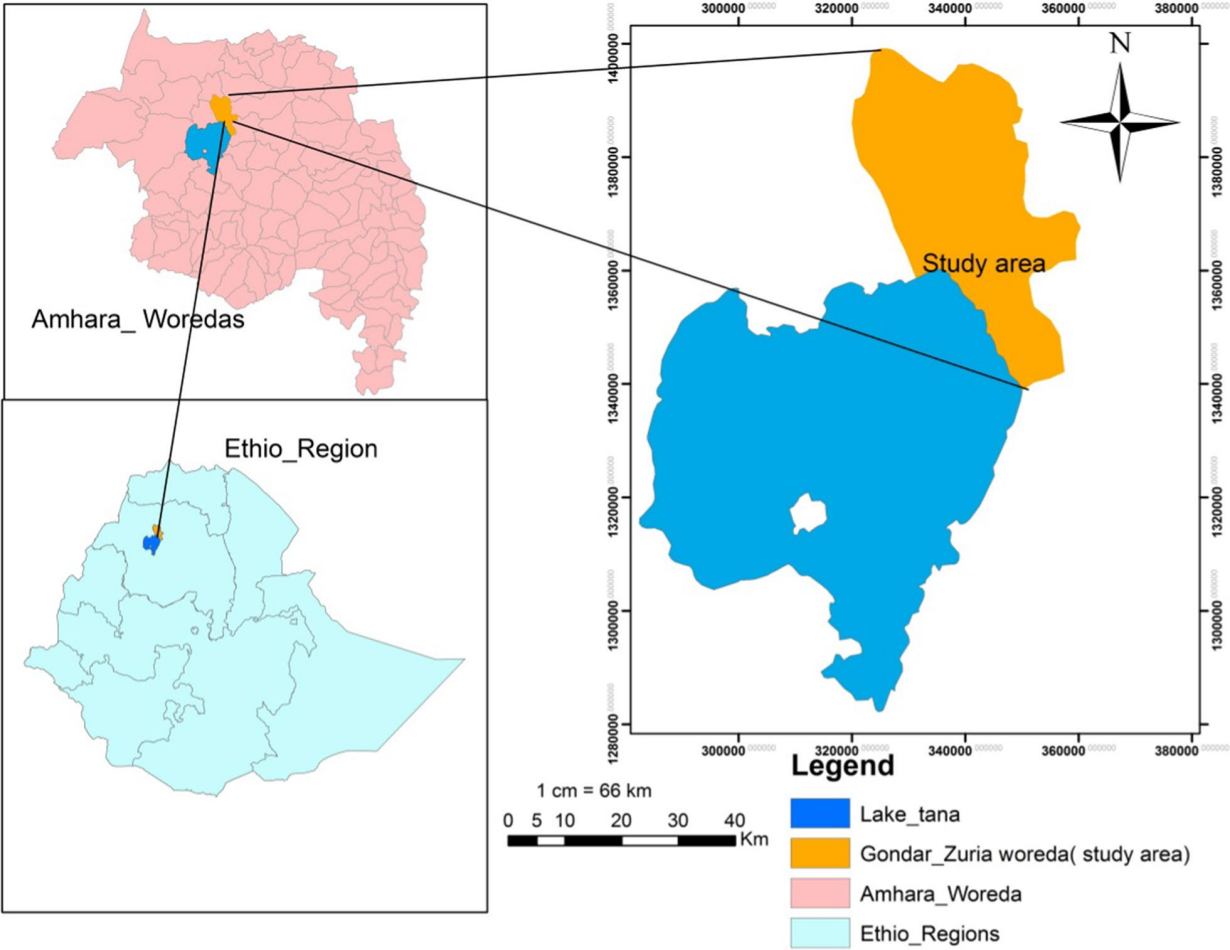
Location map of the study area and Water hyacinth status of infestation in the shore of Lake Tana (Sources: Wuletaw Mulualem)

Gondar Zuria woreda is located in the Central Gondar Zone, North Western Ethiopia. It is located on the border of Lake Tana on Northwest side with a geographical location of 12° 39′ 59.99" N Latitude and 37° 19′ 60.00′′ E Longitude. Sheha-Gomengie Kebele (Kebele-means the smallest administrative unit in Ethiopia) is about 40 km far from Gondar town and one of the 32 Kebeles found in Gondar Zuria district, was selected as a specific study area since it is one of the Kebeles with very high WH infestation. The map in Fig. 1 shows the study area in Gondar Zuria Woreda in the North Eastern Lake Tana- Sheha Gomengie Kebele.

Nowadays, the expansion rate or area coverage of water hyacinth in Lake Tana is more than its expectation; it has rapidly increased starting from its infestation period in 2011 [11]. The trend of expansion during the investigation period in 2011 was 80 ha to 100 ha [12], while in the next year (2012) it covered about 20,000 ha and in 2014 it has reached 50,000 ha [8]. A recently conducted study by [10] revealed the rapid expansion of water hyacinth, in which the area coverage of this invasive weed has increased by 50% and 82% from 2013 to 2015 and from 2015 to 2017, respectively. Currently, the Govement applied different control methods; the coverage of the weed is now being extremely decreased. The harvest weed biomass and drained nutrient load like fertilizer and other agrochemicals are the potential causes for the fast growth and expansion of water hyacinth in Lake Tana. Generally, the impact of water hyacinth is highly affecting the lake’s ecology in general and the fisheries in particular.

### Water hyacinth sample collection and transport for compost preparation

The water hyacinth was harvested manually from the shores of the Lake Tana in Sheha Kebele and transported to the composting preparing sites in 10 farmers land.

#### Treatments and experimental setup during compost preparation

**Fig. 2:**
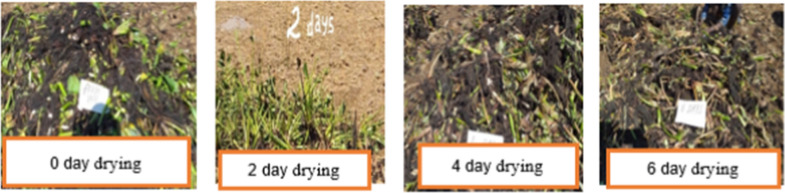
Collection of water hyacinth biomass and sun-drying process in different days

To determine better WH quality for compost preparation in terms of its drying periods, four different treatments were tested: Fresh WH (T_1_), WH sun dried for two days (T_2_), WH sun dried for four days (T_3_), and WH sun dried for 6 days (T_4_) (Fig. 2). All other materials important for compost preparation (grain straw, manure and soils) were uniformly applied in all composting heaps. To satisfy the treatment requirements, enough amounts of WH samples were collected from the lake at every two days interval as follows: the first WH sample were stayed for about 6 days being chopped and sun dried; The second WH sample were stayed for about 4 days being chopped and sun dried; The third WH sample were stayed for about 2 days being chopped and sun dried; The fourth WH sample were automatically chopped down without sun drying and used for compost preparation. The collected water hyacinth was then chopped into small pieces of using a chaff cutter to increase the surface area for microbial action. All other materials important for compost preparation (grain straw, manure and soils) were uniformly applied in all composting heap layers following the orders: 10 cm of stalk (bed); 10 cm of Water hyacinth (WH); 10 cm of grain straw; 2 cm of animal manure and finally, 2 cm soil. The above mentioned layers were repeated until the heap reached 1.5 m height and finally covered with grass to prevent water loss. Unchopped WH biomass was also used as a covering material instead of grasses.

**Fig. 3:**
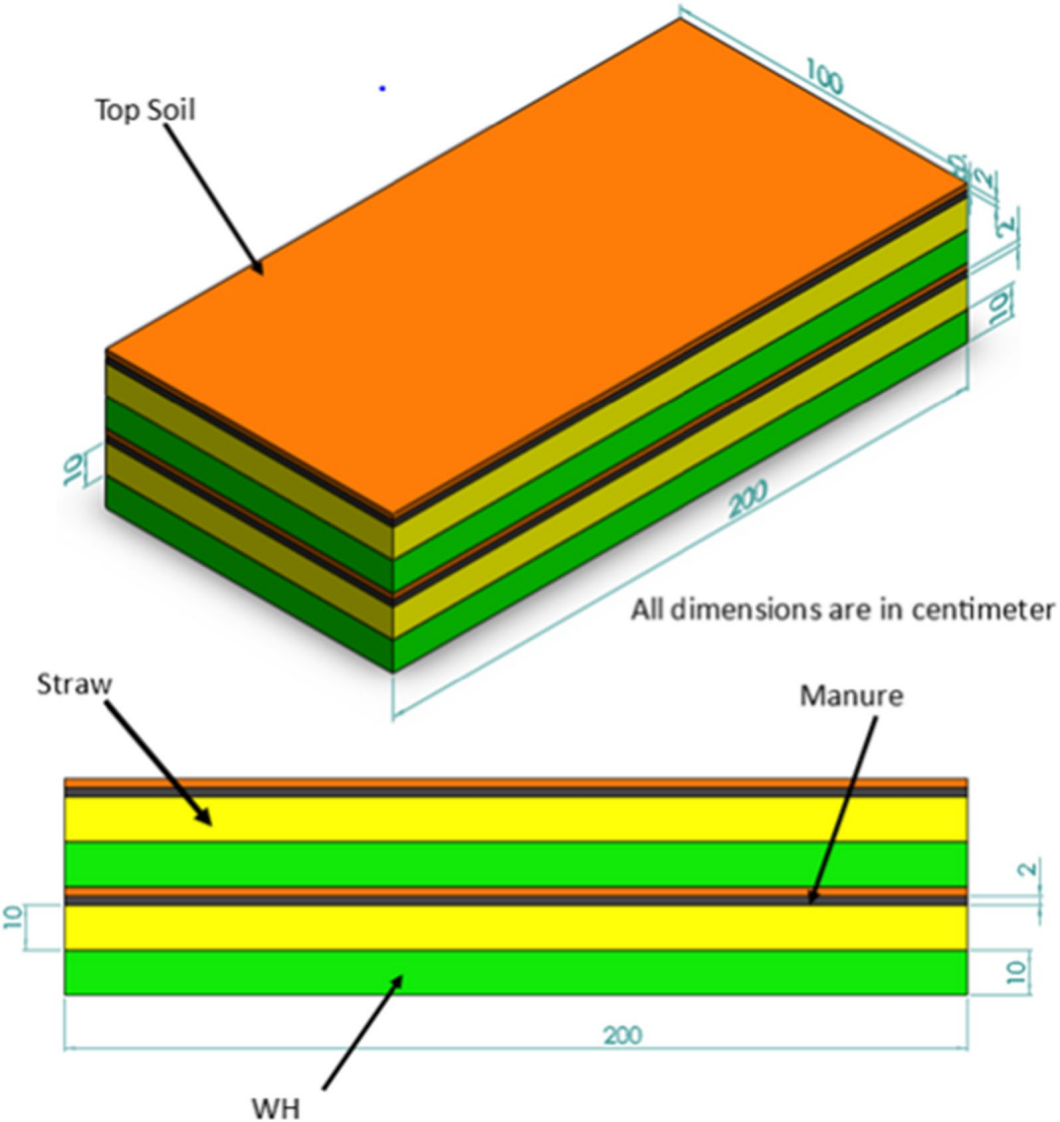
Step by step piling of composting materials (the prepared compost)

The sequences of activities performed during compost preparation were (1) Foundation pits with the size of 1 m width 2 m length and 0.2 m height were prepared; (2) Compost heaps were then constructed on the foundation pits (the heaps have 2 m length, 1.5 m height, and 1 m width) (Fig. 3); (3) to get enough space when turning over, the distances between two consecutive heaps were made sufficient (about 2 m); (4)There are 10 farmers participating in the experiment and each farmer has 4 heaps. Therefore, we have a total of 40 heaps; (5) Shades were constructed for all heaps; (6) Frequent measurements of compost temperature and height variations in the prepared compost; (7) Frequent watering of the prepared compost within three days interval for three consecutive months.

### Compost sample collection and preparation

**Fig. 4:**
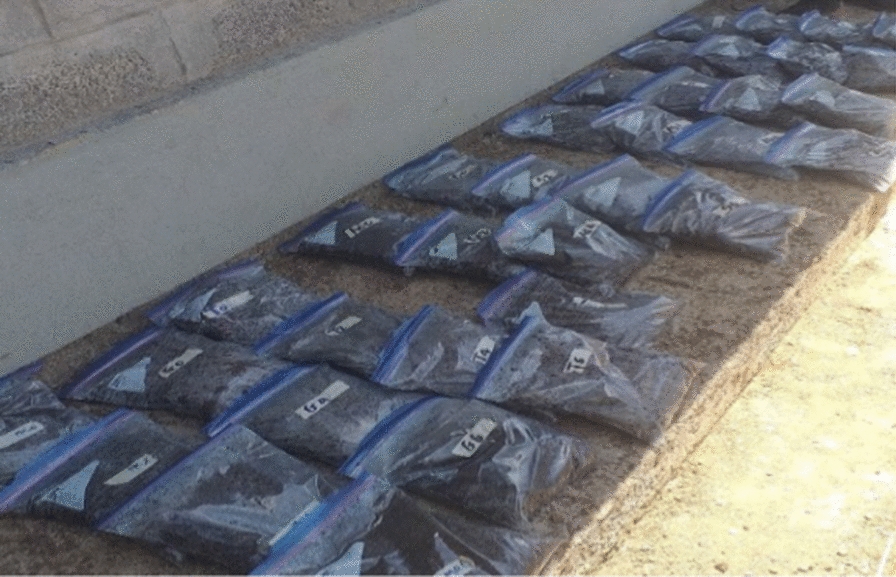
The prepared compost sample

For the experiment of utilization of water Hyacinth biomass for organic fertilizer production in Sheha Gomengie kebele, nine representative sediment samples were collected from 36 sampling sites a 500 g weight compost samples were collected from the study areas using compost sampler, and transferred into polythene bags and transported to laboratory. The compost samples that packed in plastic bags were dried in the air for 5 days in cleaned laboratory room. The air-dried compost samples were grinded with ceramic coated mortar and pestle, and passed through 2 mm sieve with in ethanol washed aluminum foil and then the analyses were done using Flam Atomic absorption spectroscopy (FAAS). Figure 4 showed the packed organic fertilizer prepared from WH.

### Instruments and apparatus

A flame atomic absorption spectrometer (FAAS) (Buck Scientific, 210VGP, USA) (FAAS) equipped with deuterium background corrector and hollow cathode lamps of Cu, Zn, Mn, Fe, Cr, Ca and Mg with air-acetylene flame was used for the analyses of metals compost samples. Polyethylene bags were used for sample collection, sample drying, preserving the grinded and homogenized samples. Electrical grinder (IAK—WERKE, Germany) was used for grinding the samples. Digital analytical balance (± 0.0001 precision) was used to weight the sample. Refrigerator (Hitach LR902T, England) was used to keep the digested and filtered samples until analyses. Borosilicate flask (100 mL) and hot plate (SH_3_, STERILINE LTD, UK) to digest the dried and powdered compost samples. Whatman filter paper no. 42, flasks (25, 50 and 100 mL), magnetic stirrer and micropipettes were used. The apparatuses used throughout the experiment were soaked with a mixture of H_2_SO_4_ and K_2_Cr_2_O_7_ for 24 h, followed by rinsing with distilled water and all the measurements were carried out at room temperature.

### Reagents and chemicals

The chemicals and reagents used in this study were all analytical grade. That was (69%) HNO_3_ (Bluluxlaboratories, Haryana, India), ammonium acetate (Bluluxlaboratories, Haryana, India), ammonium chloride (Bluluxlaboratories, Haryana, India), ethanol (98%, India) and HF (Blulux, India) used for the digestion of compost. Standard solutions of concentration (1000 mg/L) of metals Cu, Zn, Mn, Fe, Cr, Pb, and Cd (Merck, Germany) were used for preparation of calibration standards and in the spiking experiments. Distilled water was used throughout the experiment for sample preparation, dilution and rinsing apparatus prior to analysis. H_2_SO_4_ (98%) and K_2_Cr_2_O_7_ were used to prepare chromic acid solution for soaking and washing digestion flasks and other glassware before starting digestion to remove metals and other greasy contaminants left on the surface of the apparatuses.

### Analytical methods for physicochemical analyses

Compost moisture content was analyzed using the standard oven-drying method [13]. For the determination of compost heap temperature, field thermometer was employed.

#### pH

The pH of compost samples were measured using a pH meter by taking a sub-sample 5 g of air-dried compost sample and transferred into a bottle and then 25 ml of deionized water was added..

#### Cation exchange capacity (CEC) by the ammonium acetate method

An air-dried, sieved compost sample is soaked overnight with ammonium acetate. It is then filtered and leached three times with ammonium acetate, three times with 1 N ammonium chloride, and once with 0.25 N ammonium chloride, and finally washed with isopropyl alcohol. The filter cake is then leached with 225 mL of acidified NaCl.

#### Electric conductivity (EC)

For the determination of EC values of the compost samples, the compost samples were diluted with H_2_O using a mass ratio of 5:1 (water: dry compost) and shacked mechanically for 20 min, measure EC values.

#### Organic carbon content

Organic carbon content was determined using wet digestion method [14, 31].

Total N of the compost was determined through digestion, distillation and titration procedures of the Micro-Kjeldahl method [15].

#### Available P

The Olsen method was recommended for available P extraction [16].

### Total heavy metal digestion procedures

One gram of water hyacinth compost sample was placed in a 250 mL digestion tube and 50 mL of concentrated nitric acid was added [17]. The sample was then heated for 45 min at 90 °C and the temperature was increased to 150 °C at which the sample was boiled until a clear solution was obtained. Concentrated nitric acid was added to the solution (5 ml added three times) and digestion was left to take place until the volume was reduced to about 5 ml. The interior walls of the tube were washed down with distilled water and the tube was swirled throughout the digestion to keep the wall clean and prevent the loss of the sample. After cooling, 5 ml of 1% nitric acid was added to the sample. The solution was filtered with Whatman No. 42 filter paper. The solution was quantitatively transferred to a 25 ml volumetric flask by adding distilled water and analyzed using flame atomic absorption spectroscopy (FAAS) [18].

### Methods for users’ perception on WH and compost preparation

To understand farmers' perceptions, a survey questionnaire was prepared. Thirty-five farmers in the study area, of which 14 were actually participating in the research process and 21 non-participants, were selected purposively.

### Data analysis methods

The data collected was analyzed for significant differences (P < 0.05) using one-way Analysis of Variance (ANOVA) and means were separated using Tukey’s test at 5% level. All statistical analyses were undertaken using SPSS Version 20. Descriptive statistics were used to analyze the survey data.

## Results and discussion

### Physicochemical properties

The analysis of variance results of studied parameters revealed that there is no statistical difference (P > 0.05) among all studied treatments (Table 1). However, small numerical variations were observed in almost all studied parameters.

**Table 1:** Some physicochemical properties of the matured WH compost

Variables	N	T_1_	T_2_	T_3_	T_4_
pH (unit)	9	7.62 ± 0.20	7.67 ± 0.29	7.72 ± 0.26	7.71 ± 0.17
EC(ds/m)	9	2.78 ± 0.54	3.51 ± 0.97	3.34 ± 0.96	3.09 ± 0.95
CEC (cmol + /kg)	9	44.07 ± 3.18	44.57 ± 3.07	43.86 ± 2.56	46.63 ± 4.92
Moisture content (%)	9	38.71 ± 0.68	45.74 ± 1.47	46.46 ± 3.66	49.64 ± 9.07

*T*_*1*_ treatment 1 (fresh water hyacinth); *T*_*2*_ treatment 2 (2 days dry water hyacinth); *T*_*3*_ treatment 3 (4 days dry water hyacinth); *T*_*4*_ treatment 4 (6 days dry water hyacinth); *n* no of replications

#### Temperature

**Fig. 5:**
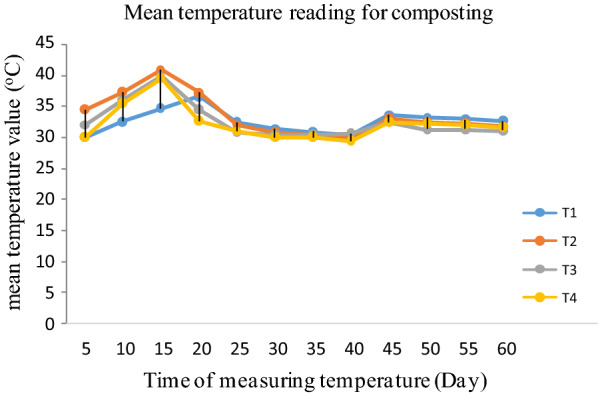
Variations in compost heap temperature with different reads

Subsequent temperature readings of the prepared compost heaps showed that comparatively lower temperature readings (30 °C) was observed in T_1_ and T_4_ in a reading taken just two days after the making of the heap. On the other hand, almost similarly higher (around 40 °C) heap temperature was attained within a week time by almost all heaps except T_1_, whereby fresh water hyacinth was used (Fig. 5). This might be related to the excess moisture contained in the fresh WH and its related microbial activity hampering roles. As the composting days increased further, a quick dropdown and then a steady decline in heap temperature was observed. Addition of optimum amount of cattle manure to the composting materials may fasten the composting process by providing easily available carbon to the microorganisms present in the composting mass. Therefore, microbial metabolic activities have increased and temperature was increased in very short time [19, 20].

#### Moisture content

Moisture contents in all treatments were comparatively less than the most reported values by different authors. In the current study, the following moisture contents were reported: 38.71 ± 0.68, 45.74 ± 1.46, 46.46 ± 3.66 and 49.64 ± 9.06(^o^C), for the fresh WH, 2 days dry WH, 4 days dry WH and 6 days dry WH, respectively (Table 1). However, the moisture content was found to be within an acceptable range of 40–50% [21]. Moisture loss during the composting process can be viewed as an index of decomposition rate, since heat generation which accompanies decomposition drives vaporization or moisture losses [22].

#### pH measurements

The pH measurements of the matured compost ranged from 7.62 ± 0.20 to 7.72 ± 0.26 (Table 1). This result is in agreement with the compost quality standards for compost used in agriculture in Switzerland (pH < 8.20) and Great Britain (7.50–8.50). The composting pH depends on the source materials and varies in each phase of the process (from 4.50 to 8.50). According to [14], the ideal pH range for most compost is from 5.80 to 7.20 [23].

#### Electrical conductivity (EC)

The mean EC values of all the treatments of matured composts ranged from 2.78 ± 0.54 to 3.51 ± 0.97 ds/m (Table 1). The result is in agreement with the recommended values reported by Mona (2003) as cited by [24], which is within 2–6 ds/m and [25], which is below 10 ds/m. The possible reasons for resulted less amount of EC at the matured phase of composting could be the volatilization of ammonia and the precipitation of mineral salts [25, 26]. For the improvement of agricultural soils, the acceptable level of EC in compost is < 4 dS/m [27, 28]. Therefore, the prepared compost is good for improving agricultural soil fertility. On analyzing the results by ANOVA, there was not significantly among all sampling sites (P < 0.05).

#### Cation exchange capacity (CEC)

The CEC value results ranged from 43.86 ± 2.56 cmol + /kg to 46.63 ± 4.92 cmol + /kg (Table 1). The presented CEC results are indicators of good decomposition rates of the WH composts. However, the results are slightly lower than the values reported by [27], which is 60%. This might be related to the differences in raw materials used for compost preparation and other management related and environmental differences.

#### Total nitrogen (TN, %)

The TN values of the matured composts ranged from 0.42 ± 0.38 to 0.75 ± 0.19 on a dry weight basis. The results are in conformity with recommendations for TN in compost by the Ontario Ministry of the Environment (2004), which recommended the typical minimum concentration (% dry weight) of Total N as 0.6. Similarly, [23] indicated that compost application having a TN concentration ranging from 0.3 to 1.5% (on dry weight basis) was considered the growth engine of the plant because it is involved in all major processes of plant development [23]. A good nitrogen supply was also indicated as important for the absorption of other nutrients [23]. Moreover, some other authors also indicated typical total nitrogen levels of finished compost to range from 0.5 to 2.5% [20, 21]. Because of the expected dynamism of the soil applied N containing fertilizers with respect to a change in the surrounding environment, one cannot predict which mineral N forms (ammonium-nitrogen, nitrate-nitrogen, and nitrite-nitrogen) will be abundant at a certain period of time. To increase agricultural production from the shore of the lake, farmers are encouraged to use manure or inorganic nitrogen fertilizers. Nitrogen requirements in the soil are usually higher as compared to other major soil nutrients for sustainable food production [27]. Therefore, our N level analysis was limited in determining the TN contents. However, as the agricultural activity in the study area is predominantly rain-fed and as the reduced soil conditions are more prevalent in these rainy seasons, nitrate-nitrogen predominance in the soil and its subsequent leaching losses is not expected in the study area.

#### Organic carbon (OC, %)

The organic carbon results ranged from 7.59 ± 1.39 to 9.1 ± 2.16 on a dry weight basis (Table 2). The result is quite low as compared to the research done in Bahir Dar which resulted in 16.6% OC of matured compost [29].

**Table 2:** The TN, OC and P contents of matured WH compost

Variables	N	T_1_	T_2_	T_3_	T_4_
Total N (%)	9	0.65 ± 0.12	0.75 ± 0.19	0.71 ± 0.23	0.75 ± 0.20
O.C (%)	9	7.59 ± 1.38	8.74 ± 2.25	8.23 ± 2.71	9.1 ± 2.16
P (g/kg)	9	2.90 ± 0.22	2.94 ± 0.14	2.74 ± 0.19	2.81 ± 0.25
C:N	9	11.60 ± 0.0	11.60 ± 0.01	11.60 ± 0.01	11.60 ± 0.00

#### Carbon to nitrogen ratio (C:N)

The C:N ratio results for all the treatments are similar (11.6) (Table 2). This result is in agreement with the finding by [32] which ranged from 10 to 11. Moreover, the result is also within range of the Ethiopian Federal EPA guidelines that recommended the C/N ratio of good quality compost to have a final C:N ratio of 29:1 or less [30].

#### Available phosphorus (P, g/kg)

High amount of P concentrations were observed in all compost treatments which ranged from 2.74 ± 0.19 to 2.94 ± 1.41 g/kg. The result is in agreement with the identified optimal 1–10 g per kg P range suggested by [23]. Available Phosphorus plays an important role in energy transfer, so it is essential for the efficiency of the plant physiological activities. Moreover, the available phosphorus concentration was increased from 2.74 to 2.93 mg/kg, in T_2_ and T_3_, respectively. The concentration of available phosphorus was increased might be due to phosphorus released by micro-organisms through the mineralization of organic matter. The results of the present study were similar to other studies [19, 20].

Phosphorus is scarce in most natural or agricultural soils and so is in the current study area. Therefore, the observed soil P deficiency in the study area can be improved by the mass production and application of P rich WH composts.

### Total heavy metal analyses

The experimental data on mean total concentrations of total heavy metals such as Cr, Mn, Zn, Cd, Cu, Fe, and Pb in the compost samples collected from 36 sampling trials in nine farmers is shown in Table 3. In the present investigation, the concentration of total heavy metals was observed with no significant difference for mean values of analysis of variance at (*P* < 0.05) (Table 3). The concentrations of metals in the compost of all treatments were below the permissible limit of different agencies [31–34] (Table 3). This result is considered as soil fertilizer with good quality compost. The result is in agreement with the standards to ensure safe application of compost laid down in Municipal Waste Management and Handling Rules notified by the Ministry of Environment and Forest, Government of India [31] and the Canadian Council of Ministers of the Environment [32, 35].

**Table 3:** Nine measurements of mean ± SD (mg/Kg) values of total heavy analysis from matured WH compost samples

Val	n	T_1_	T_2_	T_3_	T_4_	[31]	[32]	[33]
Cr	9	3.87 ± 1.95	3.53 ± 2.33	3.40 ± 0.95	3.71 ± 0.32	210	300	150
Cd	9	1.34 ± 0.15	1.31 ± 0.04	1.28 ± 0.01	1.28 ± 0.01	3	20	1.9
Zn	9	12.30 ± 4.84	14.34 ± 5.91	11.92 ± .64	12.67 ± 0.35	700	2500	140
Fe	9	35.68 ± 5.34	47.77 ± 2.96	45.32 ± 3.21	44.57 ± .52	–	–	–
Mn	9	2.85 ± 0.68	3.34 ± 0.94	6.05 ± 0.44	2.83 ± 0.69	–	–	–
Cu	9	3.39 ± 0.63	3.85 ± 0.87	6.36 ± 0.68	3.37 ± 0.64	400	500	75
Pb	9	2.13 ± 1.35	1.56 ± 0.19	1.70 ± 0.35	1.59 ± 0.24	150	500	15

#### Users’ perception on WH, compost preparation and its application

Farmers’ perception and previous experience on the use of compost and its preparation procedure are believed to be very relevant to ease WH compost production with farmers and disseminating the technology to the community. Hence, we tried to investigate farmers’ understanding and awareness of compost and its preparation procedure by interviewing 35 farmers in the study area, of which 14 were participating in the research process and 21 of them were non-participants. The questionnaire was related to farmers’ perception of WH problems, techniques of eradication, use of WH compost and preparation challenges, their experience, and future strategies to eradicate WH in the Lake Tana.

Of those interviewed farmers, all were found aware of the adverse effect of WH perhaps more than any other outsiders. They explained that the weed had adversely affected fishing, animal health, land use, and seawater utilization in their order of importance. They also added that fishing cooperatives have been abolished due to the WH invasion which has considerably been reducing the volume of fish and causing difficulty in fishing due to net damage. Animal health and its products have been affected in different ways. Milk taste has been changed to bitter, meat has become tasteless, cow dung hasn’t been suitable for firewood, and animals’ body weight has reduced significantly. It was also found that land around the lakeshore was being used for different purposes. During the dry season, the lakeshore was being used for crop production and/or grazing land. However, after the invasion of WH, the lakeshore had been completely covered by the weed and they couldn’t use the land which had a considerable benefit for them. Farmers were also using the Lake water for irrigation. However, the weed has blocked irrigation practices by making discharging water from the lake difficult as well as invading the irrigable lakeshore. The lake has been also used for drinking for animals and humans, swimming, cloth washing, and transporting services. However, the weed has been blocking such important services.

Given farmers’ understanding of WH damage, the remedy they proposed to eradicate the weed was scanty. Had the government and the people of the country as a whole not been involved in WH eradication champion, 26 percent of interviewed farmers replied that they were hopeless and starting to do nothing, and the remaining replayed to continue weeding by hand and by mobilizing the surrounding farmers although the possibility of eradication was weak. Almost all farmers (97%) interviewed believed that for the time being the problem of WH had been solved. However, 79 percent of them responded that unless continuous weeding using machine and human labour by the government or any other concerned body is carried out, the problem would exist shortly. Some other respondents (18%) suggested that planting fruits, vegetables and other appropriate plant varieties like papyrus and bamboo around the Lakeshore could be an alternative resolution to reduce WH invasion. Although some farmers understood that WH had had benefits in terms of compost preparation materials, inputs for homemade equipment, and for animal feed at a time when alternative animal feed diminish, all farmers preferred to eradicate the weed completely.

Of all farmers interviewed, 38 percent had past experience in compost preparation from non-WH materials. According to their explanation, some of the problems related to non-WH compost preparation were high labor cost, health risks during the preparation procedure, and most importantly shortage/high opportunity cost of preparation materials like grasses, weeds, leaves, and other inputs which could be used for animal feed. Some of the respondents also responded that in some cases adding compost to the soil could increase weeds and others added that the effect of compost on the fertility of the soil was not immediate and that disappointed farmers.

We also tried to compare compost from WH and other materials in terms of benefits and preparation procedures. For this purpose, those farmers who were participating in the research project were selected. They explained that the advantages of compost preparation from WH were the availability of WH, the most important input, the possibility to prepare in dry/slack season where labor is abundant, and no health risk, unlike other compost preparation procedures. Hence, of those who were participating in the compost preparation experiment, all of them vowed to prepare compost from WH for their own use without any precondition.

Finally, the investigation indicated that although farmers do have a good understanding of compost preparation procedures, all respondents have used entirely chemical fertilizer for crop production. They explained the reason that compost preparation requires a large volume of inputs perhaps unavailable to collect. Secondly, it demands hard work and a massive labor force for collecting inputs, preparation procedures and using outputs. With such a high opportunity cost of compost preparation, they confirmed that it is almost impossible to cover all cropland fertility improvement through compost. Hence, they have used chemical fertilizer which is easy to use and can cover large cropland areas though not cheap.

## Conclusion and recommendation

The results of physicochemical measurements and concentrations of nutrients are recommendable for soil fertility. Analyses of heavy metal concentrations are below the permissible limit recommended by different agencies. Therefore, the prepared compost can be recommended as it leads to significantly higher yields. Considering farmers’ understanding of compost preparation as an opportunity, converting WH into compost is promising in terms of its rich supply and the possibility of preparing it in the dry season where labor is abundant. Therefore, it can be one way of sustainably reducing WH adverse effects on the Lakeshore. Since the high-water content in WH biomass does not significantly affect the composting processes, freshly cut WH biomass can be used for compost preparation.

## Data Availability

All data generated and analyzed are included within this research article.
